# Redo accessory pathway ablation in the pediatric population

**DOI:** 10.1007/s10840-021-01064-1

**Published:** 2021-11-22

**Authors:** M. Cecilia Gonzalez Corcia, Graham Stuart, Mark Walsh, Cristina Radulescu, Francesco Spera, Maxime Tijskens, Hein Heidbuchel, Andrea Sarkozy

**Affiliations:** 1 Department of Paediatric Cardiology, Bristol Royal Hospital for Children, University Hospitals Bristol NHS Foundation Trust, Upper Maudlin Street, Bristol, BS2 8BJ UK; 2 Department of Cardiology, University Hospital Antwerp and University of Antwerp, Antwerp, Belgium

**Keywords:** Pediatric arrhythmias, WPW, Accessory pathway, Failed ablation

## Abstract

**Background:**

Literature reports 5% of recurrence/failure in paediatric accessory pathway ablations. Our aim was to investigate the reasons underlying this finding and share techniques to obtain long-term success.

**Methods:**

Thirty-nine paediatric patients referred for a repeat procedure were analysed: characteristics of the pathways and the initial and redo procedures were identified.

**Results:**

Mean age was 11.9 ± 3.3 years (59% males). Three patients (8%) had multiple accessory pathways. The most frequent location was left lateral (26%). Left sided pathway recurrence was caused mainly by poor contact (60%) and inadequate mapping (40%). For right lateral accessory pathways, poor contact accounted for 70% of failures. For antero-septal and para-Hisian locations, the use of cryoablation and choice of low radiofrequency energy delivery accounted for > 75% of failures. Long-term success strategies included choice of contact force catheters and radiofrequency applications at the ventricular insertion of the pathway and in the aortic coronary cusps. In postero-septal substrates, the main reason accounting for failure was deep or epicardial location of the pathway (37%), solved by using an irrigated tip catheter or applying lesions within the coronary sinus, or applications from both right and left postero-septal areas.

**Conclusion:**

Acute failure and post-procedure recurrence in paediatric accessory pathway ablations have multiple reasons related to the characteristics of the pathway and the technology available. Accurate understanding of the anatomy, careful mapping and pacing manoeuvers, and incorporation of new technologies contribute to achieve a definitive success in > 98% of procedures.

## Introduction

Since the first reports in the early nineties, catheter ablation has become the first therapeutic choice for re-entrant arrhythmias in children and adolescents [1–3]. Paediatric literature demonstrates a long-term success rate of 95% [4], equivalent to that of the adult patients [5–9]. Some of the factors leading to prolonged procedures, procedural failure, or recurrence of arrhythmias in the young age group have been briefly discussed [10, 11].

The aim of the present study was to investigate the incidence and underlying reasons for failure or recurrence of accessory pathway catheter ablations and determine which inherent patient’s or procedural characteristics are associated with high risk of recurrence. The initial and repeated procedures were compared in terms of techniques and strategies to help understand what may have determined failure or recurrence, and the contributions and strategies resulting in a long-term success.

## Methods

The present study has been approved by the Institutional Ethics Committee and has been performed in accordance with the ethical standards as laid down in the 1964 Declaration of Helsinki and its later amendments or comparable ethical standards.

### Study population

From 2012 to 2018, 911 young patients (< 18 years old) underwent a catheter ablation of an accessory pathway at two international tertiary electrophysiology centres (Antwerpen University Hospital, Antwerpen, Belgium, and Bristol Royal Hospital for Children, Bristol, UK). Patients with an acute failure or successful ablation with subsequent recurrence were included in this retrospective study.

### Procedure

Procedures were performed under general anaesthesia and were started by a detailed diagnostic electrophysiology study aimed to determine the arrhythmia substrate and evaluate its characteristics. The specificities in terms of technique, catheter choice, source of energy, use of 3D guided technology, and ablation settings differed depending on each individual case, resources availability, and expert’s preference. For the cryoablation procedures, the catheter used was Freezor Xtra™ (8FR, 6 mm tip) (Medtronic Inc., Minneapolis, MN, USA). For the radiofrequency procedures, the catheter of choice in > 80% of the procedures was a bidirectional DF Thermocool Smarttouch catheter (Biosense Webster, Diamond; Bar, CA, US). A common criterion was set to define immediate success after ablation including the following: (1) absence of anterograde conduction at baseline in the case of WPW, (2) adenosine test resulting in bidirectional block, and (3) absence of substrate with different protocols of progressive bi-atrial and bi-ventricular pacing at baseline and during an Isoprotenerol infusion.

### Study design

This retrospective study was performed reviewing the procedural reports of patients undergoing a repeat ablation. Data collection included patients’ characteristics at initial and redo procedures, number and characteristics of previous ablations, type and location of accessory pathway, type of access, type of catheter and energy used, type of imaging to guide the procedure, and procedure duration. The most probable reason for recurrence was determined by the operator based on the prior reports and on the findings during the redo procedure. It was particularly challenging to accurately determinate the initial reasons for failure and/or recurrence. Individual analysis of data in patient’s records was performed in order to classify initial as accurately as possible the reason for failure/ recurrence into 4 categories: (1) inaccurate mapping or diagnosis; (2) inadequate lesion formation due to poor contact, deep/epicardial location, or inadequate energy delivery; (3) inadequate long-term lesion consolidation due to the use of cryo-energy as source of energy; (4) unknown (in the case the cause remained unclear) and/or there is an inaccuracy regarding the endpoint of the assessment protocol. The differences between the initial procedure and the final procedure were compared. Recurrence was defined as reappearance of anterograde accessory pathway conduction or documented supraventricular tachycardia. Long-term success was defined as no recurrence of accessory pathway conduction, no documented supraventricular tachycardia, or palpitations during at least 24 months of follow-up after the redo procedure.

### Statistical analysis

Continuous variables are expressed as mean ± SD, or median and interquartile range (IQR). Two-sided unpaired Student’s *t*-test was used to compare continuous variables that satisfied the normality assumption. Non-parametric Wilcoxon test was used for data that deviated from normality. Categorical and binary variables are presented as frequencies (percentages). Chi-square and Fisher’s exact test were used to compare the frequency distribution of categorical variables. Analysis of variance (ANOVA) was used to analyse the differences among group means. Two-tailed values of *P* < 0.05 were considered statistically significant. The IBM SPSS Statistic version 25 software was used for the statistical analysis (SPSS, Chicago, IL).

## Results

### Patient characteristics

Thirty-nine patients (23 males, 59%) had a repeated accessory pathway ablation. Of them, 17 (43%) patients had their prior ablation in another centre. The recurrence rate after acutely successful ablations for patients that had both procedures at the participating institutions was 3.5%.

The mean age at first ablation was 11.9 ± 3.3 years, and the mean weight was 42.6 ± 14.7 kg. Thirty-one (79%) patients had structural normal hearts as assessed by transthoracic echocardiogram, 4 (10%) had Ebstein’s anomaly of the tricuspid valve, and 1 (3%) had congenitally corrected transposition of the great arteries. The remaining 3 patients (8%) had minor structural defects (1 small atrial septal defect, 1 unroofed coronary sinus, and 1 persistent left superior vena cava draining to the coronary sinus).

### Pathway characteristics

We identified 43 accessory pathway connections in the 39 patients. Three patients (8%) had multiple accessory pathways. The classification used for accessory pathway localisation was from the Working Group of Arrhythmias from the Cardiac Nomenclature Study Group [12]**.** The patient and procedure’s characteristics according to the location of the accessory pathway are summarized in Table 1. Table 2 compares the initial and redo procedures according to the location of the accessory pathway.

**Table 1:** Patient and procedure characteristics according to pathway location (continuous variables are expressed as mean, median, and first and third quartiles are displayed in the total column)

		Left Lateral (*n* = 10, 26%)	Mid-septal and para-Hisian (*n* = 8, 21%)	Postero-septal (*n* = 8, 21%)	Right lateral (*n* = 7, 18%)	Right anterior and antero-septal (*n* = 6, 21%)	Total (*n* = 39)	*P* value
Anterograde only		1	3	2	3	3	12	
Retrograde only		6	1	0	0	0	7	
Bidirectional		3	4	6	3	3	19	
Initial Procedure	N RF ablations	9 ± 4	4 ± 1	14 ± 7	11 ± 5	7 ± 2	8 (4, 15)	0.48
	Ablation time (min)	3 ± 1	2 ± 0.5	6 ± 2	8 ± 2	4 ± 1	6 (3, 9)	0.39
	Fluoroscopy time (min)	26 ± 13	23 ± 12	26 ± 11	28 ± 18	18 ± 6	22 (13, 37)	0.78
	Procedure time (min)	203 ± 86	215 ± 93	289 ± 140	248 ± 93	163 ± 57	220 (173, 280)	0.24
	Irrigated-tip catheter	2 (20%)	0 (0%)	1 (12%)	3 (40%)	0 (0%)	6 (15%)	0.35
	Energy used	RF 10	RF 5 cryo 6	RF 8 cryo 2	RF 6 cryo 1	RF 5 cryo 1	RF 34 cryo 10	0.76
	3D mapping	1 (10%)	1 (12%)	4 (50%)	2 (28%)	1 (17%)	7 (18%)	0.28
	Acute success	7 (70%)	6 (75%)	8 (100%)	7 (100%)	4 (67%)	32 (82%)	0.85
Redo Procedure	N RF ablations	10 ± 6	9 ± 3	10 ± 5	21 ± 8	9 ± 4	11 (6, 16)	0.25
	Ablation time (min)	11 ± 5	8 ± 3	4 ± 1	9 ± 4	5 ± 2	5 (3, 9)	0.25
	Fluoroscopy time (min)	22 ± 13	13 ± 3	10 ± 3	18 ± 8	14 ± 4	15 (9, 25)	0.63
	Procedure time (min)	203 ± 72	199 ± 46	241 ± 144	233 ± 96	232 ± 70	203 (139, 289)	0.93
	Irrigated-tip catheter	5 (50%)	4 (50%)	4 (50%)	4 (57%)	1 (17%)	18 (46%)	0.45
	Energy used	RF 9- cryo 3	RF 6- cryo 4	RF 8- cryo 3	RF 7	RF 6-cryo 1	RF 36-cryo 11	0.92
	3D mapping	5 (50%)	6 (75%)	5 (62%)	5 (71%)	4 (67%)	24 (61%)	0.74
	Acute success	10 (100%)	8 (100%)	8 (100%)	7 (100%)	5 (83%)	38 (97%)	0.77
	Long-term success	10 (100%)	8 (100%)	8 (100%)	6 (86%)	5 (83%)	37 (95%)	0.87

*Cryo*, cryoenergy; *min*, minutes; *n*, number; *RF*, radiofrequency energy

**Table 2:** Comparison of initial and redo procedures according to pathway location (continuous variables are expressed as mean)

	Left lateral (*n* = 10)			Mid-septal para-Hisian (*n* = 8)			Postero-septal (*n* = 8)			Right lateral (*n* = 7)			Right anterior and antero-septal (*n* = 6)		
Procedure	Initial	Redo	*P* value	Initial	Redo	*P* value	Initial	Redo	*P* value	Initial	Redo	*P* value	Initial	Redo	*P* value
N RF ablations	9 ± 4	10 ± 6	0.82	4 ± 1	9 ± 3	0.57	14 ± 7	10 ± 5	0.33	11 ± 5	21 ± 8	< 0.01	7 ± 2	9 ± 4	0.59
Ablation time (min)	3 ± 1	11 ± 5	0.12	2 ± 0.5	8 ± 3	0.28	6 ± 2	4 ± 1	0.22	8 ± 2	9 ± 4	0.62	4 ± 1	5 ± 2	0.91
Fluoroscopy time (min)	26 ± 13	22 ± 13	0.50	23 ± 12	13 ± 3	0.17	26 ± 11	10 ± 5	< 0.01	28 ± 18	18 ± 8	0.51	18 ± 6	14 ± 4	0.64
Procedure time (min)	203 ± 86	203 ± 72	0.99	215 ± 93	199 ± 46	0.70	280 ± 140	241 ± 144	0.51	248 ± 93	233 ± 96	0.82	163 ± 57	232 ± 70	0.34
Irrigated-tip catheter	2 20%	5 50%	0.16	1 12%	4 50%	0.10	1 12%	5 50%	0.19	3 40%	4 57%	0.59	0 0%	1 17%	0.90
RF	10 100%	9 90%	0.82	5 62%	6 75%	0.59	8 100%	8 100%	1	6 85%	7 100%	0.84	5 83%	6 100%	0.82
3D map	1 10%	5 50%	0.14	1 12%	6 75%	0.01	4 50%	5 62%	0.61	2 28%	5 71%	0.10	1 17%	4 67%	0.24
Main reason for failure/recurrence	IM 5 PC 3 DL 2			CR 6 LP 2			DL 3 PD 2 PC 2 U 1			CR 1 PC 5 U 1			IM 1 PC 2 LP 2 CR 1		

*cryo*, cryoenergy; *min*, minutes; *n*, number; *RF*, radiofrequency energy

For initial reason for failure, *IM* stands for inaccurate mapping; *PC*, inadequate lesion formation due to poor contact; *DL*, inadequate lesion formation due to deep location; *PD*, inadequate power delivery (inside the coronary sinus); *LP*, limited choice of radiofrequency power; *CR*, use of cryoablation; *U*, unknown cause

The fluoroscopy times for the initial procedure had a median of 22 min [13], which is similar to the timing reported in some adult studies [14]. The length of the fluoroscopy can be accounted for the characteristics of the substrate, the expertise of the operator (being both centres teaching units with constant involvement of trainees in the procedures) and the limited access to 3D mapping technology for standard cases (10%). Redo procedures included higher use of 3 D mapping (50%) and probably completely or earlier involvement of experts in the field. These factors probably contributed to the reported reduction in the fluoroscopy time to 15 min [9].

The initial ablation failed in 7 patients (18%), 70% (*n* = 5) in an external institution.

### Left lateral accessory pathways

#### Initial procedure

Ten patients (26%) presented for a repeat procedure for a left lateral accessory pathway. In all cases, the initial ablation was performed via trans-septal access. The choice of a trans-septal approach was performed on individual electrophysiologist’s preference as per his/her regular practice. In 3 cases (30%), the accessory pathway could not be eliminated during the procedure. The most frequent reason for a repeat procedure was inadequate mapping (*n* = 5, 50%), followed by poor contact (*n* = 3, 30%).

#### Repeat procedure

On average, the redo procedure was performed 1.5 years after the initial intervention (10.9 ± 3.6 vs 12.4 ± 1.9 years). In all cases, the redo ablation was performed via trans-septal access due to interventionist’s first choice. Strategies for long-term success at the redo procedure included incorporation of a steerable long-sheath (80%), choice of an irrigated tip-catheter (50%), and 3D electro-anatomical mapping (50%).

### Para-Hisian and mid-septal accessory pathways

#### Initial procedure

In the case of para-Hisian and mid-septal accessory pathways (*n* = 8, 21%), 2 patients (25%) had failure in the initial procedure and 6 (75%) had recurrence of accessory pathway conduction. In all, the main reason for repeated procedure was inadequate lesion formation due of cryoablation (*n* = 6, 75%) or the use of low power radiofrequency energy (*n* = 2, 25%).

The mean cryoablation application time was 13.2 min; the initial procedure was successful in eliminating the pathway, but recurrence occurred within 1 month.

In the 2 patients that had a radiofrequency ablation, it was abandoned due to high risk of AV node injury after an application time < 2.5 min.

#### Repeat procedure

**Fig. 1:**
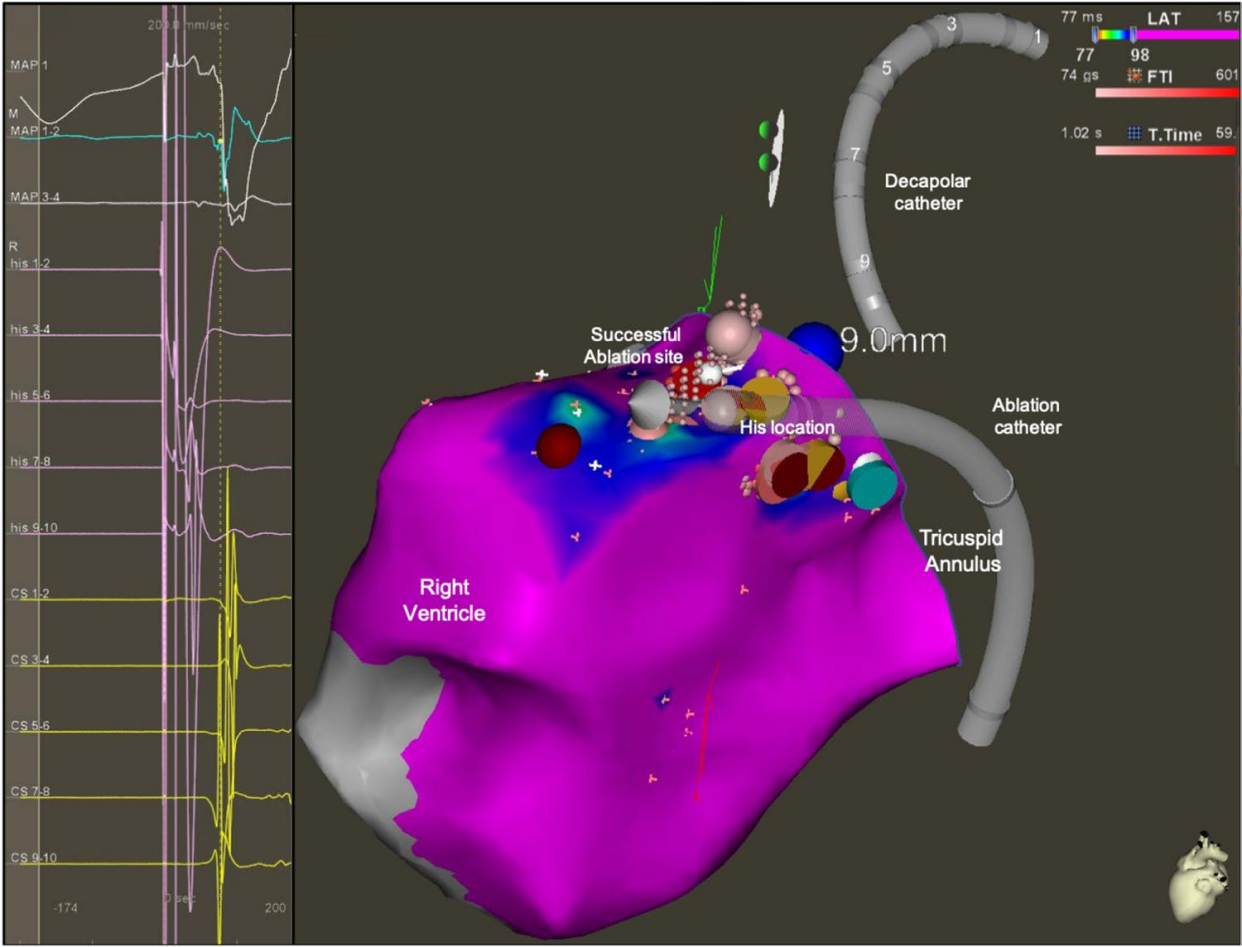
3D electro-anatomical activation map of a para-Hisian accessory pathway successful ablated at the right ventricle insertion. On the left side: surface electrocardiogram (ECG) and intracardiac electrograms (EGM) recording the earliest activated signal of the right ventricle during right atrium pacing in a patient with pre-excitation. On the right, 3D reconstructions of the tricuspid valve and right ventricle with activation map during atrial pacing. The reduced activation window shows a gradient of colours from red (earliest activated site) to purple (latest activated site). The red dot shows the successful ablation site, 9 mm distant from the site where we produced mechanical AV block with high contact force > 20 g (blue dot). The yellow dots show the site where the His potential was recorded

**Fig. 2:**
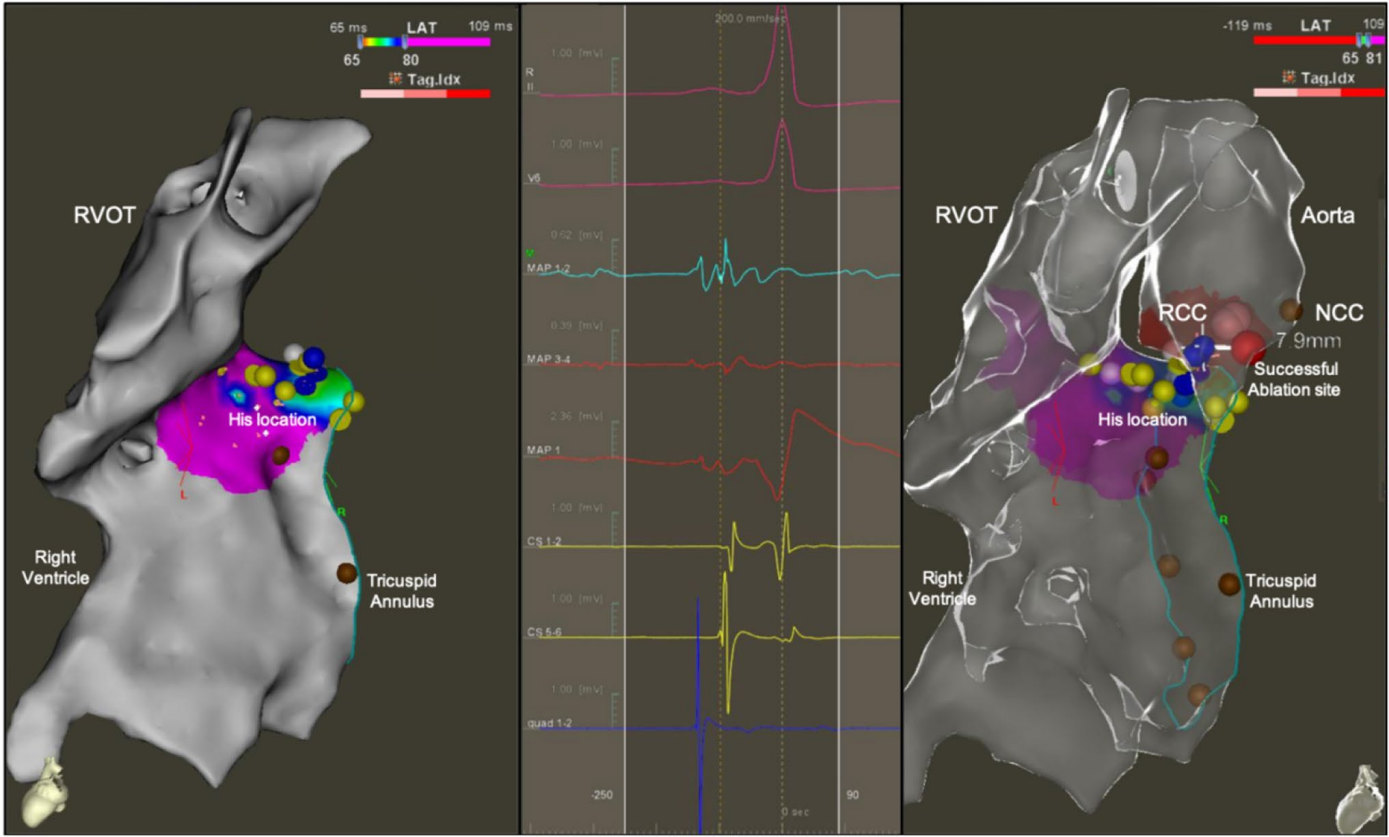
3D electro-anatomical activation map of a para-Hisian accessory pathway successfully ablated from the right coronary cusp of the Aorta during a redo ablation procedure. Seven-year-old patient referred after two failed cryoablations and frequent episodes of tachycardia despite bi-therapy with antiarrhythmics drugs. During the electrophysiology study, a bidirectional antero-septal accessory pathway with easily inducible orthodromic tachycardia was identified. After meticulous mapping of the septal region and identification of the earliest signals in the His region and unsuccessful ablation at close proximity of this site, aortic root mapping was performed. The earliest ventricular signal on the ablation catheter was identified in the right coronary cusp. Aortic root angiogram was performed and a distance of 10 mm was measured between the right coronary artery orifice and the target site. A radiofrequency ablation was performed using an energy of 20 W with temperature of 50 °C, with immediate and definitive termination of the accessory pathway conduction within 4 s. The left and right panels show the electro-anatomical reconstruction of the right ventricle, right outflow tract, tricuspid annulus (brown dots), and aortic root with an activation map of the accessory pathway earliest signals on the ventricular insertion (red area). The yellow dots show the areas where His potentials were recorded; the red dots show the successful ablation site located 7.9 mm from the site where mechanical AV block was induced with high contact force (> 20 g) at the tip of the ablation catheter (blue dots). Within the non-coronary cusp (NCC brown dot), we identified large atrial electrograms. The middle panel shows the surface ECG and intracardiac EGMs recorded on the successful ablation site in the right coronary cusp (RCC) during sinus rhythm, with a clear sharp accessory pathway signal on the distal ablation catheter

A mean of 2 years elapsed between the initial and redo procedures (13.2 ± 2.1 vs 15.2 ± 4.4 years). All redo ablations were performed with radiofrequency, and in 75% 3D mapping was chosen to guide the procedure (Figs. 1 and 2).

### Postero-septal and coronary sinus accessory pathways

#### Initial procedure

**Fig. 3:**
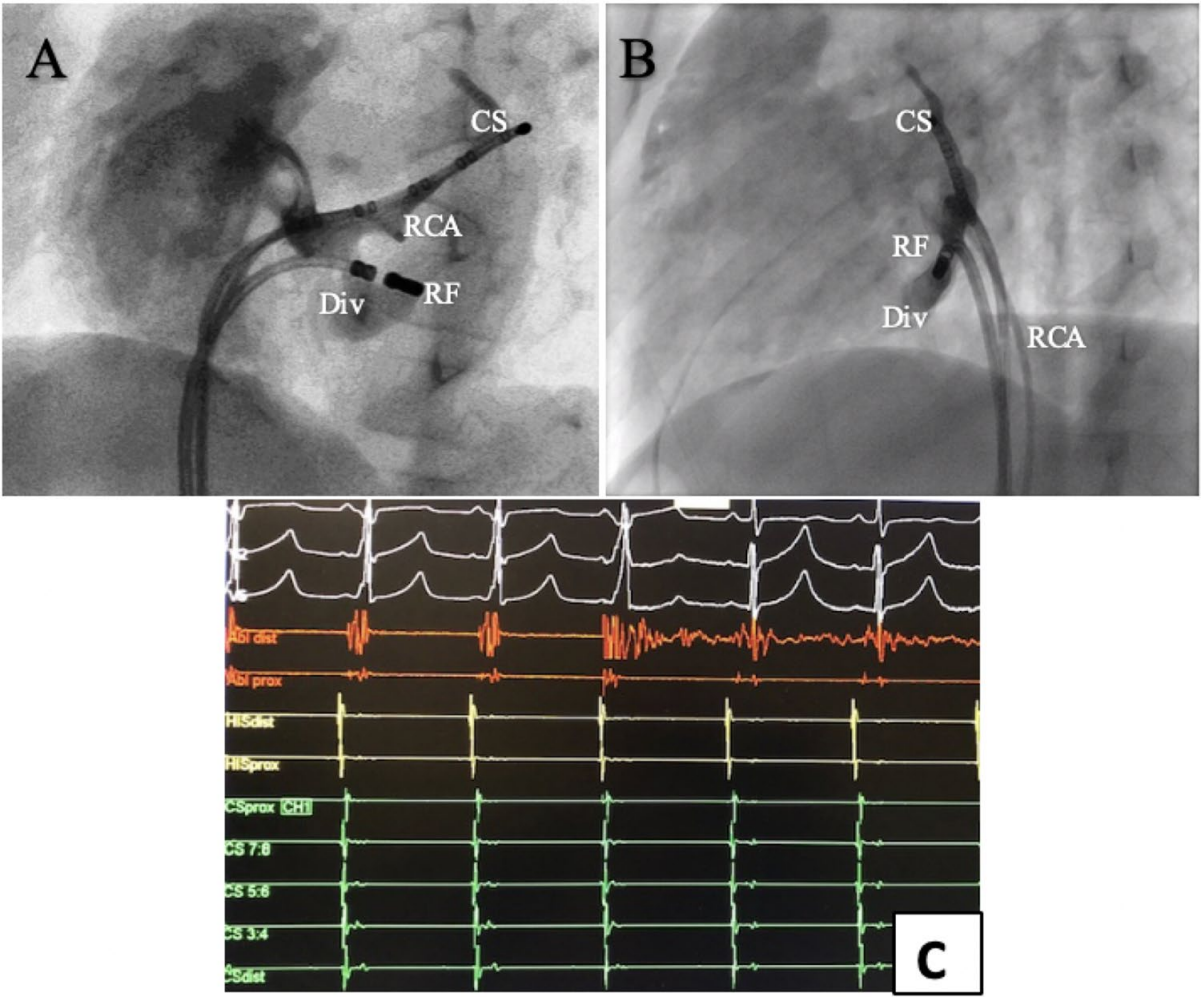
Repeated ablation of a bidirectional accessory pathway within a diverticulum of the coronary sinus in a 9-year-old patient presenting with pre-excited atrial fibrillation. **A**, **B** RAO and LAO fluoroscopic projections during a coronary sinus angiogram performed via pump-injection through a right coronary artery angiogram catheter (RCA). The radio-frequency ablation catheter (RF) is placed inside the diverticulum via right femoral vein access. A decapolar catheter is placed in the main body of the coronary sinus (CS). **C** Intra-cardiac ECMs during the successful radiofrequency ablation with an irrigated-tip catheter. The pathway was immediately eliminated once the radiofrequency lesion started

Postero-septal accessory pathways (*n* = 8, 21%) presented with the highest number of non-successful applications and the longest procedure length. Three patients (38%) had pathways located deep in the septal area requiring bi-atrial applications. Two patients (25%) presented with a diverticulum of the coronary sinus (Fig. 3). The cause leading to recurrence in these cases was probable multiple including lack of lesion depth, epicardial location, and poor delivery of energy when lesions were located within the coronary sinus.

#### Repeat procedure

The redo procedures implemented long sheaths (*n* = 5, 63%) and irrigated-tip catheters (*n* = 4, 50%). The second procedure was performed at an average of 3 years post-initial ablation (11.5 ± 3 vs 14.6 ± 1.9). The source of energy was radiofrequency in all the redo procedure, and 3 (38%) incorporated irrigated-tip catheters.

### Right lateral accessory pathways

#### Initial procedure

All patients presented with post-procedure recurrence (*n* = 7, 18%), with 3 (42%) involving Mahaim fibres. The main reason for recurrence seemed to be inadequate lesion formation due to lack of catheter stability in 5 (72%). Interestingly, a long sheath was used in only 1 (14%) case during the initial procedure while an irrigated catheter was preferred in 3. Overall, this group experienced the longest fluoroscopy times and ablation times.

#### Repeat procedure

**Fig. 4:**
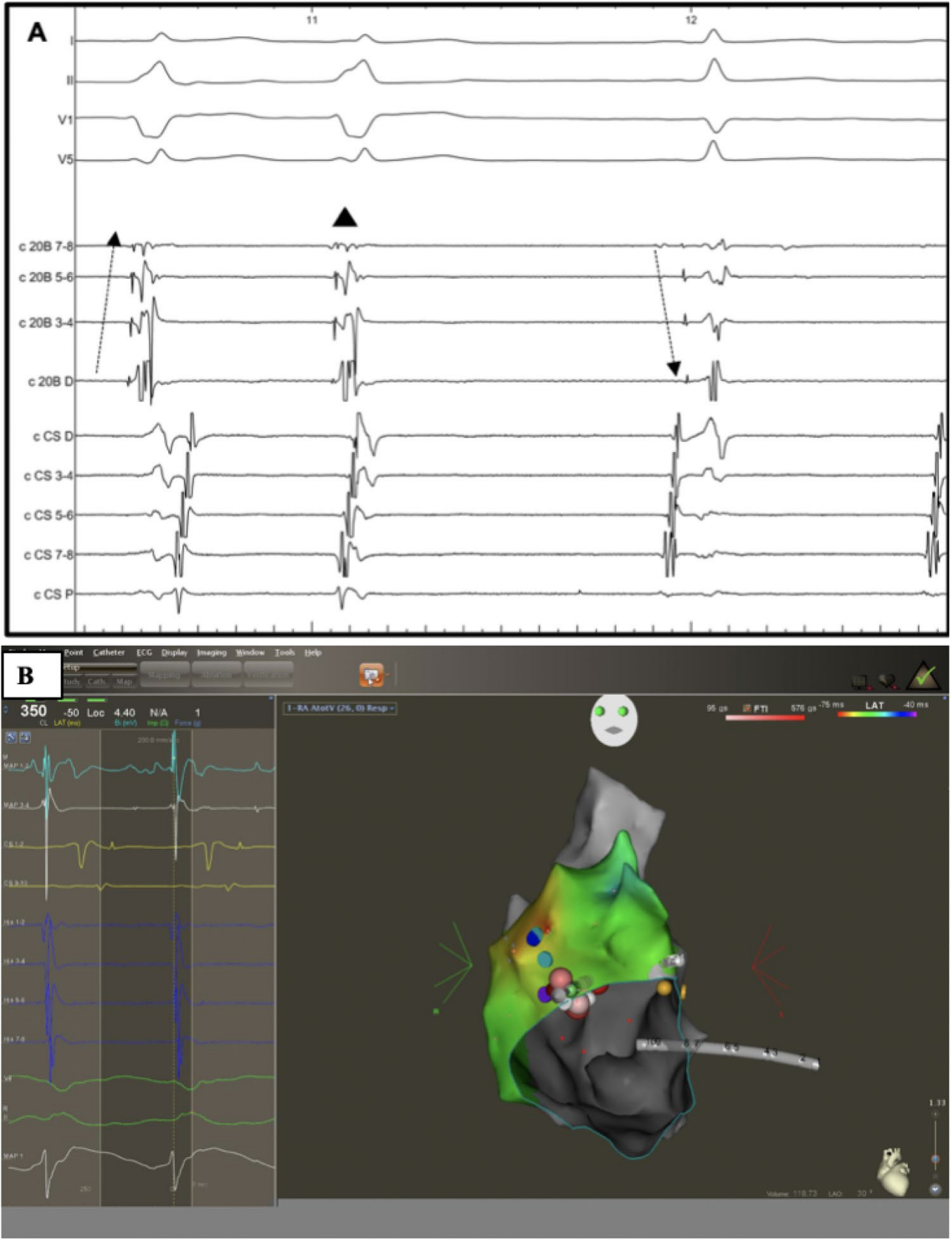
Surface ECG (electrocardiogram) and intracardiac EGMs (electrograms) and 3D electro-anatomical activation map in a young patient with a Mahaim accessory pathways during the repeated ablation procedure. **A** Patient with antidromic atrio-ventricular re-entrant tachycardia (AVRT) and two previous failed catheter ablation procedures. The successful ablation site 5 with 3D electroanatomical mapping was between 10 and 11 o’clock of the tricuspid annulus. On the first beat during tachycardia, the His activation is from distal to proximal (dashed arrow) with a short HV interval, probing an antidromic mechanism. On the second beat, a spontaneous atrial premature activation interrupts the tachycardia (black triangle). On the third beat during sinus rhythm, there is evidence of reversal of the His activation to proximal to distal (dashed arrow) activation with a normal HV interval. **B** 3D electro-anatomical and activation map reconstruction of the right atrium and tricuspid valve during antidromic atrio-ventricular re-entrant in the same patient. The yellow dots show the areas where His potentials were recorded; the green dot demonstrates the area of the Mahaim potential, which was also the site of the successful ablation (red dots)

**Fig. 5:**
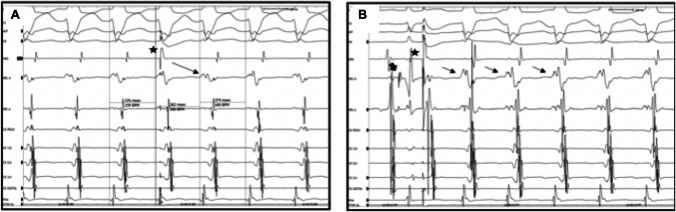
Surface ECG (electrocardiogram) and intracardiac EGMs (electrograms) in a young patient with a Mahaim accessory pathways during the repeated ablation procedure. **A**, **B** Intracardiac electrograms of young patient with a repeated procedure for a Mahaim fibre. The successful ablation was performed conventionally under fluoroscopy with 4 mm non-irrigated tip catheter, with applications between 8 and 9 o’clock on the tricuspid annulus. Panel A shows the tracings during antidromic atrioventricular re-entrant tachycardia (AVRT) with an atrial premature beat (APB) on His refractoriness (black star) from the HRA catheter (positioned at the lateral wall of the right atrium) which pre-excites the next ventricular activation with continuation of tachycardia. **B** An antidromic AVRT initiation by a paced single atrial premature beat from high right atrium (black star) with the ablation catheter positioned in the His region. Notice the reversal of His activation sequence from proximal to distal activation in sinus rhythm (black big arrow) to distal to proximal His activation (small black arrows) at initiation and during antidromic AVRT

The redo-procedure was performed 1.1 year after the initial procedure (13.2 ± 3.5 years, and 14.3 ± 4.3 years). During the redo procedures, a combination of long sheath and radiofrequency energy was selected in all cases. Moreover, in 4 cases (57%), irrigated tip catheters and 3D mapping were selected. The redo ablation was acutely successful in all cases. Figures 4 and 5 depict two examples of patients with Mahaim accessory pathways.

### Right anterior and antero-septal accessory pathways

#### Initial procedure

Four patients (67%) had a recurrence and 2 (33%) had a failed initial procedure. The main reasons leading to a repeated procedure seemed to be poor contact (*n* = 2, 33%) and limited application (*n* = 2, 33%) due to proximity of the AV node. Overall, this location correlated with the lowest energy delivery and the lowest mean temperatures (mean power 25.3 ± 8.6 W and mean temperatures 47.7 ± 6.6 °C).

#### Repeat procedure

There was a mean of 2 years between the first and second ablation (10.4 ± 4 vs 12.5 ± 4.4 years old). At the redo-procedure, a successful outcome was achieved using a long sheath (*n* = 4, 67%) and/or an irrigated-tip catheter (*n* = 5, 83%).

### Recurrence following a second procedure and multiple (≥ 3) procedures

Our experience showed that after a second procedure, a 99% success rate can be achieved in expert’s hand and with optimal technology. Six (15%) patients presented ≥ 3 procedures for the initial substrate that recurred on multiple occasions. Two patients remained with unsolved substrates (an antero-septal pathway and a right lateral pathway).

### Complications

One patient (2%) presented with a small self-resolved pericardial effusion following a trans-septal access. Two patients (4%) presented with superficial small hematomas at the puncture site. Specifically, we confirm that no adverse events occurred in younger patients with weight < 20 kg due to incompatibility between the catheter and the patient’s size/weight.

## Discussion

Our study corroborates that the long-term success of accessory pathway ablation in the paediatric population can be increased to > 98% after a repeated procedure. It also demonstrates that the main reasons for failure include inaccurate mapping and/or diagnosis, and inadequate long-term lesion formation (mainly related to the use of cryo-energy during the initial procedure).

Even if many expert consensus on management of accessory pathway in the paediatric age estimate the single procedural acute success at 94–95% [11–13], this figure is not universally accepted. Some studies have shown higher recurrence rates when an extended follow-up period is evaluated [10, 15]. Thus, a significant number of patients require multiple procedures to achieve definitive success. A recent review of risk factors associated with recurrence in the paediatric population suggests that the position and number of accessory pathways are related to higher incidence of recurrence following ablation [15]. Our group has also identified specific challenges at each position and built a body of hypothesis that may be associated with recurrence. These risk factors are still pending validation with larger studies involving an appropriate methodology.

The main apparent reasons for the recurrence especially in the left and right lateral location were related to difficulties with catheter stability, which can be solved with the introduction of contact force, the use of 3D electro-anatomical mapping and long sheaths, and direct visualization of the interface catheter-tissue with intracardiac echography imaging.

It has been long apparent that left lateral pathways are the most frequent [14]**.** Regardless of the location, a deep understanding of the anatomic landmarks and a careful mapping seem to be the keys for success. A right lateral position correlates with more difficult procedures with higher number of lesions to achieve success, leading to incorporate long (preferably steerable) sheaths, contact force sensing catheters, and in rare cases, jugular access to assure better catheter stability. Moreover, a common condition that eventually makes ablation difficult in Mahaim fibres is the temporary disappearance of the pathway conduction and extreme susceptibility to mechanical block.

Likewise, Ebstein’s anomaly is a risk for failed ablation and recurrence, the difficulties being related to the high prevalence of multiple pathways and to the lack of catheter stability secondary to the displacement of the tricuspid valve [16, 17].

Para-Hisian and antero-septal pathways’ challenge relates to the proximity of the AV node. The wisdom of an accurate mapping and catheter stability cannot be overemphasized. Some centres advocate for the use of cryoablation to avoid injury to the nodal structures, advocating that stability is achieved once the ice ball is formed facilitating contact. However, an important drawback is that the catheter is stiff which interferes with manipulation in small hearts. Given the high prevalence of recurrence with cryo-energy (15–20%) [18, 19] it has been our choice to attempt a cautious radiofrequency ablation protocol including a conscious delineation of the right atrium, tricuspid valve, and right ventricle anatomy with 3D activation mapping. Rotational angiography of the right atrium with 3D overlay on live fluoroscopy has been recently incorporated in many laboratories to help a precise location of the His position. Because the His bundle is located deeper and protected with a fibrous tissue envelope in the ventricular septum as compared to the atrial and superficially located compact AV node, a key point with this approach is to target the ventricular insertion by searching for predominant ventricular signals with a small atrial. In the challenging cases of high-risk anterograde only accessory pathways with no inducible supraventricular tachycardia, mapping is usually performed during fast atrial pacing in an attempt to maximize ventricular pre-excitation. In the case of bidirectional pathways in this location, both atrial (in case of inducible orthodromic reciprocating tachycardia) and/or ventricular mapping during fast atrial pacing or sinus rhythm can be used. In all cases, we emphasize on confirmation of underlying intact AV node conduction with differential atrial pacing maneuvres before and during the ablation. In some cases, the use of contact force technology, carefully increasing the contact from 5 to above 30 g allows precise identification of the sites of both mechanical AV block and mechanical bumping of accessory pathway conduction. The 3D contact force catheters currently available in the market are irrigated tip catheters (ThermoCool SmartTouch catheter, Biosense Webster, CA, USA, and TactiCath™ Quartz Contact force ablation catheter, Abbot, Chicago, IL, USA). Our strategy has been to set the irrigation mode low (usually 2 to 7 ml/min) during ablation, to use them as non- or minimally irrigated catheters and thus limit the extension of the lesion. In selected cases, applications with limited energy delivery (starting at 5 to 10 W and escalating by 5 W until 25–30 W) allow highly accurate lesions, which can be stopped immediately in the case of junctional acceleration and/or AV block [20]. However, the drawback is that this strategy can result in local oedema thus reducing direct contact between the catheter and the fibre. The use of a long (preferably steerable) sheath, a superior approach via the jugular vein, and performing the ablation during apnea may also increase catheter stability and help to limit the risk for AV node injury.

An alternative approach for antero-septal accessory pathways is mapping and targeting the accessory pathway from the aortic cusps. This approach can be safely performed with adequate precautions and may be considered in cases with failed previous procedures and/or high risk of AV block with the standard right-sided approach [21, 22].

In the case of postero-septal pathway ablations, an irrigated-tip catheter may help target deep myocardial or epicardial substrates [23], rarely requiring combined right and left atrial applications, or intracoronary applications. Because coronary sinus aneurysms are also related to long and difficult procedures and high incidence of recurrence, we advocate for very thorough understanding of the anatomy using an angiogram, 3D electro-anatomical mapping, or intracardiac echocardiography.

## Conclusions

Recurrent accessory pathway ablation in children remains a challenge even in expert’s hand. There is inherent logic to adapt the procedure in terms of energy used, catheter choice and mapping strategy depending on the location of the pathways. Technology improvements have been impressive in recent years and collaborate to improve procedure outcomes. However, the cornerstones of successful and safe paediatric accessory pathway ablation remain a deep understanding of the anatomic landmarks, meticulous mapping including pacing strategies when necessary, and catheter stability with adequate energy delivery during applications.

## Limitations

This is a retrospective observational study without a matched control group, which limits the strength of many observations. We acknowledge that the reason for a substrate recurrence may have been oversimplified in some patients.
